# Acute Pain and Development of Opioid Use Disorder: Patient Risk Factors

**DOI:** 10.1007/s11916-023-01127-0

**Published:** 2023-07-01

**Authors:** Livia Baumann, Corina Bello, Filipovic Mark Georg, Richard D. Urman, Markus M. Luedi, Lukas Andereggen

**Affiliations:** 1 Department of Anaesthesiology and Pain Medicine, Kantonsspital Aarau, Aarau, Switzerland; 2 Faculty of Medicine, University of Bern, Bern, Switzerland; 3 Department of Anaesthesiology and Pain Medicine, Inselspital, Bern University Hospital, University of Bern, Bern, Switzerland; 4 Department of Anesthesiology, The Ohio State University, Columbus, OH USA; 5 Department of Anaesthesiology and Pain Medicine, Cantonal Hospital of St, Gallen, St. Gallen, Switzerland; 6 Department of Neurosurgery, Kantonsspital Aarau, Aarau, Switzerland

**Keywords:** Acute pain, Opioid crisis, Opioid use disorder, Patient risk factors

## Abstract

**Purpose of Review:**

Pharmacological therapy for acute pain carries the risk of opioid misuse, with opioid use disorder (OUD) reaching epidemic proportions worldwide in recent years. This narrative review covers the latest research on patient risk factors for opioid misuse in the treatment of acute pain. In particular, we emphasize newer findings and evidence-based strategies to reduce the prevalence of OUD.

**Recent Findings:**

This narrative review captures a subset of recent advances in the field targeting the literature on patients’ risk factors for OUD in the treatment for acute pain. Besides well-recognized risk factors such as younger age, male sex, lower socioeconomic status, White race, psychiatric comorbidities, and prior substance use, additional challenges such as COVID-19 further aggravated the opioid crisis due to associated stress, unemployment, loneliness, or depression.

**Summary:**

To reduce OUD, providers should evaluate both the individual patient’s risk factors and preferences for adequate timing and dosing of opioid prescriptions. Short-term prescription should be considered and patients at-risk closely monitored. The integration of non-opioid analgesics and regional anesthesia to create multimodal, personalized analgesic plans is important. In the management of acute pain, routine prescription of long-acting opioids should be avoided, with implementation of a close monitoring and cessation plan.

## Introduction

Opioid use disorder (OUD) is a pattern of harmful opioid use that leads to clinically significant distress or impairment with physical or mental impairments and/or legal consequences [1••]. OUD is defined as any opioid intake fulfilling at least two of the criteria listed in Table 1. Dependence and addiction both are included in the diagnosis of OUD [2]. However, it is important to distinguish those two, to reduce stigmatizing (e.g., of patients suffering from chronic pain who developed dependence, a physiologic response to long-term opioid use), enable adequate treatment of true addiction (with long-acting agonists such as methadone or buprenorphine being the only therapeutic option to lower the risk of death in opioid addicts), or to prevent unethical legal consequences for patients who are on such substitution medications (e.g., report to child welfare authorities and subsequent separation from mothers who take methadone) [3] (Table 2).

**Table 1:** Diagnostic criteria for opioid use disorder (adapted by Siddiqui [1••])

- Opioid intake in larger amounts or over a longer period of time than planned	
- Continuous desire or unsuccessful efforts to control or reduce its usage	
- Majority of time spent obtaining, using, or recovering from substance use	
- Cravings to consume opioids	
- Opioid use leading to failure in daily work or life responsibilities	
- Continued use despite social problems caused or aggravated by opioid use	
- Quitting of important activities of daily living as a result of use	
- Repeated use in situations where opioids can be physically dangerous	
- Repeated use despite knowing it can cause physical or psychological problems	
- Development of tolerance (need for rising dosage in order to experience the same effect or decreasing effect with the same amount)	
- Existing withdrawal symptoms or continued opioid use to avoid those

**Table 2:** Criteria defining dependence and addiction [84]

Dependence	Addiction
- The need for a substance in order function, e.g., also the use of insulin or anti-hypertensives - Physiologic adaptation to taking certain substances over a prolonged period of time - Withdrawal symptoms present - Generally more common	- Continued use of a substance, loss of control and strong craving despite negative consequences such as high risks compared to benefits or even harm caused by its use - Persistent epigenetic changes even after long-term abstinence - Presence or absence withdrawal symptoms

Since the 1990s, increased prescribing of opioids in high-income countries has led to iatrogenic dependence and later to illicit opioid use. Since 2010, several interventions to reduce the supply and extra-medical use of prescribed opioids, such as prescription monitoring programs and “pill mill” laws, have been introduced. These interventions led to a transition from opioid to heroin use from the late 2000s, followed by a third wave starting around 2013 as highly potent synthetic opioids like illicitly manufactured fentanyl entered the market [4–7].

Opioids are used to treat acute and chronic pain, such as post-injury pain, or chronic diseases such as cancer [1••]. In surgical patients, a new persistent opioid use is not an uncommon phenomenon and is not significantly different between a minor and major intervention, but rather associated with behavioral disorders [8]. Thereby, patients that were on opioids before a surgical procedure had a tenfold increase in the development of persistent postoperative opioid use [8, 9]. Alarmingly, previously opioid-naïve patients demonstrated persistent opioid use following surgery ranging from 2 to 6% [8, 9].

Opioids remain an important and potent measure when treating acute postoperative pain, although recent findings showed that excessive and/or prolonged prescribing, both in-hospital and at discharge, contributes to the current opioid crisis and increase patient harm [10••].

In 2017, the global age-standardized rate of opioid dependence per 100,000 people was 510, with the highest estimated prevalence in the USA at 1347 per 100,000 people [4]. In the USA, the highest rates of drug use and treatment were noted in northern areas, whereas southern states as well as nonmetropolitan areas had less OUD treatment. Still, the latter may rather point to a limited access to medical support, such as addiction or rehabilitation facilities, naloxone availability, or needle exchange programs, especially in areas with a higher prevalence of minorities [1••, 11].

Several risk factors for OUD have been identified, including younger age, male sex, lower education level, lower socioeconomic status, race/ethnicity, psychiatric comorbidities [1••, 12, 13], and prior substance use [7, 14•, 15]. Moreover, the COVID-19 pandemic aggravated the opioid crisis due to increased stress, unemployment rates, isolation and subsequent loneliness, and depression [16•].

In order to reduce OUD, opioid prescriptions should be made after evaluating both, the patient’s risk factors and individual preferences, and short-term prescribing should be considered. In addition, the opioid use should be monitored and followed closely in those patients at risk for persistent or new chronic opioid use [17••]. To minimize opioid usage, non-opioid analgesics and regional anesthesia should be used whenever possible [1••, 18–22]. Long-acting opioids should either be avoided or used with caution, as they can increase the risk of opioid use dependency [1••, 10••, 23, 24]. In addition, managing OUD should be based on psychosocial behavioral interventions, with medications such as naloxone for acute opioid overdose and methadone or buprenorphine for long-term addiction treatment [1••, 25–27].

## Recent Findings

The main risk factors for the development of OUD, such as younger age, male sex, lower education level, lower socioeconomic status, race/ethnicity, psychiatric comorbidities [1••], and prior substance use, have been identified previously [14•]. Besides, there is a lack of information about the situation in most parts of the world, as most of the present literature is centered around the current situation in the USA [10••]. Information obtained from the USA can however be used in countries where the opioid crisis has not reached US levels, but a high risk for the development of similar problems exists regarding opioid-linked dependency problems, especially as big pharmaceutical companies expand into low- and middle-income countries [10••].

In this narrative review, we capture a subset of recent advances in the field targeting the literature on patients’ risk factors for OUD in the treatment for acute pain.

### Major Risk Factors for OUD Development

A major risk factor for OUD is the duration of patients’ misuse following initial therapy for acute pain, with no fewer than 6–19 days of opioid intake increasing dependency by 3.5-fold, and 20–30 days by 5.5-fold [28•, 29, 30]. Moreover, the frequently observed intake of higher opioid doses and duration than initially prescribed correlates with the risk of OUD development [28•, 31]. After adjusting for confounders, it was observed that patients using their own prescriptions were at a greater risk of developing OUD than those who illegally bought or received opioids from family members or friends [28•]. This could be explained by the fact that access to opioids is easier when a patient is in possession of a prescription for potential misuse [28•]. Considering the number of unreported cases of patients with illegal opioid use, more research is needed to better comprehend these use patterns.

Another patient-related risk factor for OUD development in the treatment of acute pain is a history of mental illness such as anxiety disorders, or depression [10••], including the morphine’s mood-elevating effects of opioids [28•]. Moreover, patients with preexisting use of antidepressants, benzodiazepines, or other recreational drugs such as tobacco, alcohol, or cocaine are at an increased risk of developing OUD [9, 10••, 22, 32, 33].

### Postoperative, Surgical, and Trauma-Related Pain as a Risk Factor for OUD

Adequate pain management is crucial in preventing the development of OUD in the perioperative setting [34]. Pain treatment options other than opioids, such as regional anesthesia and non-opioid analgesics, should be given priority [1••]. Undertreatment of pain results in unnecessary suffering and may lead to patients seeking additional pain management. On the other hand, overprescribing of opioids can lead to prolonged use and dependency. Therefore, patients should always receive appropriate follow-up after hospital dismissal and be supported in reducing opioid use [1••].

Current evidence confirms increased risk of developing OUD in patients exposed to opioids following trauma, as early as 3 months after initial exposure [35]. Also, patients requiring multiple surgical interventions and intensive care unit (ICU) treatment were more likely to develop OUD [14•]. Therapeutic plans must be carefully considered, as lower incidence of OUD and development of chronic pain are being noted in those receiving multimodal therapy compared to those receiving opioids only [14•], including non-opioid analgesics and regional anesthesia as a base, with the aim of reducing opioid application whenever possible [1••, 14•]. Patient preferences should always be taken into account when establishing a personalized pain treatment plan [17••]. When opioid prescription is necessary, patients’ individual risks of developing OUD should be evaluated, and prescriptions as well as follow-up meetings and treatment should be coordinated accordingly [17••]. The time of opioid application should be kept to a minimal amount, and prescriptions are limited to short-acting opioids when treating acute pain [1••, 10••]. Regarding extended-release opioids, current recommendations do not support using them in the management of both acute and postoperative pain due to various reasons: they are difficult to manage due to their slow onset time, increase the risk of adverse drug events such as ventilator depression, and finally, increase the risk of persistent postoperative opioid use for longer than 30 days [10••, 24, 36–39].

### Risk Factors in Pediatric, Adolescent, and Elderly Patients

Most opioids prescribed for adults are also used by children and are generally well-tolerated, except for codeine [40••]. The latter has unpredictable side effects due to different genetic variations in the CYP2D6-phenotype resulting in altered metabolization: children and neonates breastfed by mothers taking codeine may suffer from serious adverse events and even are at risk of death [40••, 41]. Compared to opioid management in adults, children are mostly prescribed opioids in excess of their analgesic needs [40••]. However, after an uneventful pediatric procedure, they should rather provide supply for 3 to 5 days if needed at all [40••]. Caregiver education in managing analgesics is important to implement such a safe therapeutic concept, as they often underdose opioids in children following discharge from hospital [40••, 42, 43], while no evidence has been found that opioid use in children leads to later misuse and death when used for acute pain and with correct administration and prescription [40••]. Besides, caregivers frequently don’t receive instructions on safe opioid storage and disposal of eventually unused opioids [40••, 44]. Of note, opioids prescribed upon discharge have been associated with long-term opioid use in adolescents with prior chronic pain syndrome, substance abuse, or mental health issues; therefore, follow-up meetings arranged by specialized pain services are advised for these patients [40••].

In the elderly population (i.e., > 65 years of age), problematic opioid use and OUD have been increasingly observed, with both illicit and prescription opioids [45]. Risk factors for OUD development in this patient cohort include multimorbidity, long-term alcohol misuse, depression, or chronic pain disorder [45, 46]. Along with the current therapy plans established from the data of younger adults, elderly patients respond well to opioid agonist therapy, while age is no obstacle to treatment [45].

Regardless of age, however, opioid misusage has been linked to psychological factors and single social determinants of pain rather than somatic factors [46].

### Racial Disparities Regarding the Risk of OUD Development

In the management of pain and of OUD, there are significant demographic disparities encountered [1••]. Preventive interventions introduced over the last few years were targeting the White population disregarding minorities, thus leading to a higher prevalence of opioid-related death in the latter group [1••]. In addition, Black patients are more likely to have their pain undertreated and are less likely to receive opioid medication when treated for acute pain [17••, 47]. Namely, clinicians tend to falsely believe that Black patients are at higher risk for OUD [17••, 48]. Even with knowledge about patients’ preferences regarding their analgesic therapy and risk factors for substance abuse, racial disparities in treatment for acute pain remain [17••]. Additionally, Lee and colleagues noted that apart from a decreased likelihood of receiving opioids for pain relief, African-Americans and Hispanics were less likely to receive any analgesia upon presentation with acute pain at all [49•]. Although some cultural differences may confound these differences in therapy, as, for example, the value of stoicism in some African-American communities limiting adequate pain management, this situation is alarming, especially since this disparity has not changed over the last 30 years [47, 49•]. Interestingly though, the prevalence of OUD among Hispanics and the Latin population is among the highest compared to other ethnicities [1••]. Furthermore, even though minority children in the emergency department were more likely to achieve a ≥ 2-point reduction in pain on a 10-point scale, they were less likely to obtain optimal pain reduction when being treated in the emergency department [1••, 47]. Preventive interventions introduced over the last few years have targeted the White population rather than minorities, thus leading to a lower prevalence of opioid-related deaths in the former compared to the latter group [1••]. Thus, a standardized screening program and pain management protocol should be implemented in order to improve acute pain therapies and targeted interventions to reduce OUD in people of all ethnicities [1••, 50–53].

### Acute Pain Management in the COVID-19 Era and Its Sequel with Regard to OUD

As of 2019, the COVID-19 pandemic posed new challenges to the global consequences of the opioid crisis: in the USA, patients with OUD were at a higher risk of COVID-19 hospitalization and subsequent worse course of disease, including death, sepsis, kidney failure, and an increased risk of intubation [54]. Beside, an increase of non-prescribed drugs, in particular fentanyl, was noted with a major influence on the opioid crisis since the start of the pandemic [16•, 55]. Potential reasons include increased stress, job loss, loneliness, and depression related to social distancing during the pandemic, which could have aggravated the increased prescription and overuse or intake of illicit drugs, both in people initiating drug intake and people relapsing after prior use [56]. In addition, isolation made it more difficult for people to bond with others or to call for immediate help in the case of acute overdosing [56].

In general, OUD patients suffering from COVID-19 were older, more likely to be Black or Hispanic with an increased likelihood of having other medical comorbidities like hypertension, cardiovascular disease, chronic pain disorder, diabetes, substance disorders, or mental health issues [54]. However, even after accounting for those demographic characteristics and comorbidities, patients with COVID-19 infections in conjunction to OUD were at a higher risk of requiring hospitalization and experiencing medical complications compared to patients without OUD [54]. Interestingly, a few comorbidities were noted to be protective in patients with both OUD and COVID-19. Although results are conflicting [57–59], studies indicated that asthma was not associated with a higher intubation rate or higher incidence of kidney failure, sepsis, and death [54, 60]. This protective effect has been noted before in other COVID-19 cohorts without OUD, potentially being explained by changes in the viral entry receptor expression, the use of steroids, or the chronic inflammatory response [54]. Furthermore, another protective effect has been linked to chronic pain syndrome, with fewer intubations being noted in patients infected with COVID-19, as well as a lower rate of kidney failure and mortality [54]. In contrast, previous studies showed more severe COVID-19 outcomes in patients treated with long-term opioids, wherefore a firm relationship remains elusive [54].

In the COVID-19 era, approximately 22% of opioid-naïve patients were treated with opioids during hospitalization, with 8% of them receiving a new opioid prescription upon discharge [61]. In contrast, in the pre-COVID era, reported rates of a discharge opioid prescription ranged as high as 29% [61]. Potential reasons include clinicians making efforts to reduce opioid prescription in general and in COVID-19 patients in particular, for whom opioids are not recommended because of the risk of respiratory impairment [61]. Nevertheless, opioid use during hospitalization was associated with a discharge prescription, with every additional day in the hospital increasing the likelihood to receive the latter [61].

Appropriate discharge prescriptions are vital and should be consistent with the current practice guidelines of safe opioid stewardship, with close follow‐up to reduce their availability in the community [62].

### OUD Development in Headache Patients

The trend of increasing opioid prescriptions for acute pain has also been noted in patients with headache disorders, despite a lack of evidence of benefit from its use [63–66]. Opioid use as a regimen for acute migraine attacks led to an increased rate of returns to the emergency department due a lacking benefit during the acute phase [63]. Furthermore, using opioids as the initial treatment for acute headaches was associated with a longer length of hospital stay and led to a high risk of medication overuse headache [67]. Depending on the specific type of headaches, opioids should be avoided and primarily treatment should include non-steroidal anti-inflammatory drugs (NSAIDs), neuroleptic antinauseants, triptans, or corticosteroids [63]. Second, headache patients should be monitored carefully and followed up systematically in order to reduce the effect on their quality of life and the related burden on society and health care [63, 67].

Given the significant drawbacks of opioids, referral to a neurological center with headache specialists is key for patients to ensure correct diagnosis and adequate treatment planning depending on the type of headache [68]. In addition, more research is needed to establish specific protocols in the management of headache patients in the acute care setting [69–71].

### OUD Development for Acute Pain Treatment in the US Military

Members of the US military are at particularly high risk of OUD or use of other sedatives [72–74]. A 53% increase in drug overdose mortality from 2010 to 2019 has been reported [72]. In the US military, 98% of patients treated for combat-related injuries received at least one prescription for opioids, and 83% of patients received an outpatient opioid prescription [75••]. Nevertheless, only 4% of patients became sustained opioid users, although many patients who managed to quit opioid usage still had subsequent episodes of opioid misuse [75••]. Factors associated with a higher risk of opioid misuse are consistent with risk factors for the general population and included lower socioeconomic status, increased severity of the injury requiring five or more procedures during hospitalization, and comorbid mental health disorders [32, 75••, 76]. Likewise, Black soldiers were at a lower risk of overusing opioids than White soldiers [75••]. Another risk factor identified in the development of long-term opioid dependence among members of the US military included the prescription of long-acting opioids during the first 30 days of an acute pain or physical trauma, rather than using immediate-release opioids [77]. Finally, veterans may benefit from peer support to help them reduce their substance use and improve their quality of life and mental health [72]. The “buddy system” used during military service can be applied to drug use in veteran circles, where the “buddy” is trained to administer naloxone or render first aid in case of opioid-related breathing problems [72].

In summary, in the acute setting, the use of short-acting opioids is pivotal in members of the military suffering from more severe injuries, additional mental health conditions, lower socioeconomic status, or known previous opioid exposure. Moreover, these patients experience particular benefits from multimodal pain management and regular follow-up visits [72, 75••].

### Prevention Strategies for OUD Development

Prevention strategies for OUD development imply proper assessment and identification of patients at risk for opioid misuse or addiction. However, most screening tools were either based on low-quality studies or were hardly efficient to adequately identify patients at risk for opioid misuse [78]. In addition, both acute and chronic pain might not get reported by some patients, in particular by older persons, those with cognitive or mental impairment, and/or patients with substance abuse [79, 80]. Better assessment tools along with an improved monitoring system of the prescribed opioids are necessary [79, 81]. Importantly, clear communication between clinicians and patients, along with an active involvement of the latter group in the decision-making process towards optimized pain management, is necessary to minimize risks associated with opioid misuse [82]. The same approach should be used in children and adolescents, enforcing the engagement of patients themselves and their families in opioid stewardship efforts [83].

## Conclusion

In order to reduce the prevalence of OUD, opioid prescriptions should be made after evaluating both individual patients’ risk factors and preferences. In addition, short-term prescription should be considered and patients at high risk for dependence closely monitored. A shift of standards and protocols towards non-opioid analgesics and regional anesthesia as the basis for multimodal analgesic treatment plans is urgently required. In the management of acute pain, routine prescription of long-acting opioids should be avoided, with implementation of a close monitoring and cessation plan supported by pain specialists.
